# Acute heart failure presentation, management, and outcomes in cancer patients: a national longitudinal study

**DOI:** 10.1093/ehjacc/zuad020

**Published:** 2023-03-08

**Authors:** Briana Coles, Catherine A Welch, Rishabh S Motiwale, Lucy Teece, Clare Oliver-Williams, Clive Weston, Mark A de Belder, Paul C Lambert, Mark J Rutherford, Lizz Paley, Umesh T Kadam, Clare A Lawson, John Deanfield, Michael D Peake, Theresa McDonagh, Michael J Sweeting, David Adlam, VICORI collaborative, Sarah Darby, Chris Gale, Mike Hawkins, Alexander Lyon, Jem Rashbass, Raoul Reulen, Alistair Ring Adam Timmis, Sally Vernon Jennifer Lai, Paul Charlton, Akosua Donkor, Nadeem Fazal, Anil Gunesh, Andrew Harrison, Abbas Khushnood, Andrew Goodwin, Peter Ludman, Theresa McDonagh, Francis Murgatroyd, Brian Shand, Sally Vernon, David Forman

**Affiliations:** Biostatistics Research Group, Department of Health Sciences, George Davies Centre, University of Leicester, University Road, Leicester, LE1 7RH, UK; Biostatistics Research Group, Department of Health Sciences, George Davies Centre, University of Leicester, University Road, Leicester, LE1 7RH, UK; National Disease Registration Service, NHS Digital, Wellington Place, Leeds, LS1 4AP, UK; Biostatistics Research Group, Department of Health Sciences, George Davies Centre, University of Leicester, University Road, Leicester, LE1 7RH, UK; School of Medicine, George Davies Centre, University of Leicester, University Road, Leicester, LE1 7RH, UK; Biostatistics Research Group, Department of Health Sciences, George Davies Centre, University of Leicester, University Road, Leicester, LE1 7RH, UK; National Disease Registration Service, NHS Digital, Wellington Place, Leeds, LS1 4AP, UK; Biostatistics Research Group, Department of Health Sciences, George Davies Centre, University of Leicester, University Road, Leicester, LE1 7RH, UK; Department of Cardiology, Glangwili General Hospital, Dolgwili Road, Carmarthen, SA31 2AF, UK; National Institute for Cardiovascular Outcomes Research, Arden & GEM Commissioning Support Unit, St John's House, 30 East Street, Leicester, LE1 6NB, UK; Biostatistics Research Group, Department of Health Sciences, George Davies Centre, University of Leicester, University Road, Leicester, LE1 7RH, UK; Department of Medical Epidemiology and Biostatistics, Karolinska Institutet, 171 77, Stockholm, Sweden; Biostatistics Research Group, Department of Health Sciences, George Davies Centre, University of Leicester, University Road, Leicester, LE1 7RH, UK; National Disease Registration Service, NHS Digital, Wellington Place, Leeds, LS1 4AP, UK; Diabetes Research Centre, University of Leicester, Leicester General Hospital, Gwendolen Road, Leicester, LE5 4PW, UK; Department of Health Sciences, George Davies Centre, University of Leicester, University Road, Leicester, LE1 7RH, UK; Diabetes Research Centre, University of Leicester, Leicester General Hospital, Gwendolen Road, Leicester, LE5 4PW, UK; Department of Cardiovascular Sciences, University of Leicester and NIHR Leicester Biomedical Research Centre, University of Leicester, Glenfield Hospital, Groby Road, Leicester, LE3 9QP, UK; National Institute for Cardiovascular Outcomes Research, Arden & GEM Commissioning Support Unit, St John's House, 30 East Street, Leicester, LE1 6NB, UK; Institute of Cardiovascular Science, University College London, 62 Huntley Street, London, WC1E 6DD, UK; National Disease Registration Service, NHS Digital, Wellington Place, Leeds, LS1 4AP, UK; Department of Respiratory Medicine, University of Leicester, Glenfield Hospital, Groby Road, Leicester, LE3 9QP, UK; King's College Hospital, Denmark Hill, London, SE5 9RS, UK; Biostatistics Research Group, Department of Health Sciences, George Davies Centre, University of Leicester, University Road, Leicester, LE1 7RH, UK; Statistical Innovation, AstraZeneca, City House, Hills Road, Cambridge CB2 1RY, UK; Department of Cardiovascular Sciences, University of Leicester and NIHR Leicester Biomedical Research Centre, University of Leicester, Glenfield Hospital, Groby Road, Leicester, LE3 9QP, UK

## Abstract

**Aims:**

Currently, little evidence exists on survival and quality of care in cancer patients presenting with acute heart failure (HF). The aim of the study is to investigate the presentation and outcomes of hospital admission with acute HF in a national cohort of patients with prior cancer.

**Methods and results:**

This retrospective, population-based cohort study identified 221 953 patients admitted to a hospital in England for HF during 2012–2018 (12 867 with a breast, prostate, colorectal, or lung cancer diagnosis in the previous 10 years). We examined the impact of cancer on (i) HF presentation and in-hospital mortality, (ii) place of care, (iii) HF medication prescribing, and (iv) post-discharge survival, using propensity score weighting and model-based adjustment. Heart failure presentation was similar between cancer and non-cancer patients. A lower percentage of patients with prior cancer were cared for in a cardiology ward [−2.4% age point difference (ppd) (95% CI −3.3, −1.6)] or were prescribed angiotensin-converting enzyme inhibitors or angiotensin receptor antagonists (ACEi/ARB) for heart failure with reduced ejection fraction [−2.1 ppd (−3.3, −0.9)] than non-cancer patients. Survival after HF discharge was poor with median survival of 1.6 years in prior cancer and 2.6 years in non-cancer patients. Mortality in prior cancer patients was driven primarily by non-cancer causes (68% of post-discharge deaths).

**Conclusion:**

Survival in prior cancer patients presenting with acute HF was poor, with a significant proportion due to non-cancer causes of death. Despite this, cardiologists were less likely to manage cancer patients with HF. Cancer patients who develop HF were less likely to be prescribed guideline-based HF medications compared with non-cancer patients. This was particularly driven by patients with a poorer cancer prognosis.

## Graphical Abstract

**Graphical abstract:**
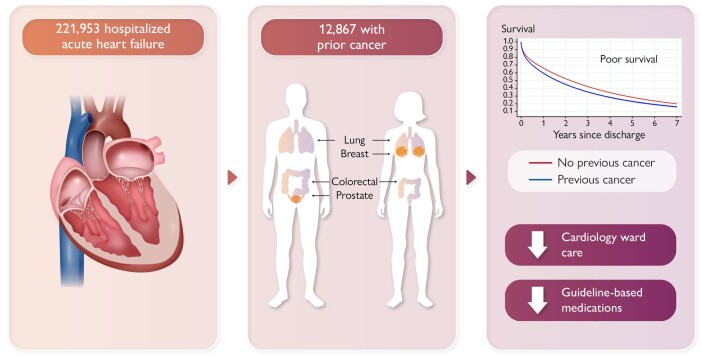

## Introduction

Survival in heart failure (HF) patients is often described as being akin to the prognosis of some cancers. However, with recent improvements in early detection and cancer treatment, patients with cancer are living longer.^1^ As a result, optimal management of co-morbidities and cardiovascular risk factors is becoming an increasingly important determinant of outcomes.^2^ Cancer survivors are at increased risk of developing cardiovascular diseases including heart failure (HF) which is associated with a poor prognosis.^3^ The association between cancer and HF is, in part, attributable to overlapping risk factors and pathophysiological pathways.^4–6^ Additionally, cancer treatments can contribute to cardiac dysfunction through chemotherapy-induced cardiotoxicity and/or chest radiotherapy.^7–9^ A multidisciplinary cardio-oncology approach is necessary to improve outcomes in cancer survivors with HF.^10,11^

Guideline-based management of HF alleviates patients’ symptoms, reduces hospital admissions, and improves outcomes.^12^ Effective treatments include angiotensin-converting enzyme inhibitors or angiotensin receptor antagonists (ACEi/ARB), beta-blockers, mineralocorticoid receptor antagonists (MRA), and, more recently, the angiotensin receptor–neprilysin inhibitor and sodium–glucose co-transporter 2 inhibitors.^12^ In cancer patients, ACEi and beta-blockers have been shown to improve cardiac function in patients with chemotherapy-induced cardiotoxicity.^9,13^ These data are currently limited to anthracycline and anti-HER2–based treatments with little data on other classes of cancer therapies such as those frequently used to treat prostate, lung, and colon cancer. Although outcome data for other HF medications in a cancer-specific population are currently lacking, it is likely the benefits on HF outcomes are no different to the general HF population. However, it is less clear whether survival in cancer patients presenting with acute HF is primarily driven by HF or cancer outcomes. There is also little existing evidence on whether cancer patients with HF receive the same care as HF patients without cancer.

The objective of this study was to compare the presentation, treatments, and outcomes between hospitalized HF patients with or without a preceding diagnosis of one of the four most common cancers in the UK (breast, prostate, colon/rectum, lung) using the Virtual Cardio-Oncology Research Initiative (VICORI) research platform.^14^ The VICORI data sets link English national cancer registry and cardiovascular audit data with hospital coding and death certification data. This provides a unique opportunity to investigate the interplay between cardiovascular diseases and cancer.

## Methods

### Ethical approval and consent to participate

This study was reviewed and approved by the VICORI Consortium Project Review Panel. The VICORI research programme has received favourable ethical opinion from the North East—Newcastle and North Tyneside 2 Research Ethics Committee (REC reference 18/NE/0123). The study was performed in accordance with the Declaration of Helsinki.

### Study design and databases

This is a retrospective, population-based cohort study using linked national cancer registry and HF audit databases. The VICORI study was approved by the UK Health Research Authority and the National Research Ethics Service (18/NE/0123). This study was reviewed and approved by the VICORI Consortium Project Review Panel and the National Disease Registration Service (NDRS) Project Review Panel.

The National Heart Failure Audit (NHFA) collects information on adults with an unscheduled (non-elective) admission to a hospital in England and Wales who have a death or discharge with a diagnosis of HF in the primary position (ICD-10 code I11.0, I25.5, I42.0, I42.9, I50.0, I50.1, or I50.9).^15^ The National Disease Registration Service compiles a comprehensive, quality-assured data set referred to as the National Cancer Registration Dataset (NCRD); this is collated using a wide range of data sources to register all tumours diagnosed for residents of England.^16^ Until recently, pseudonymized cardiovascular audit and cancer registry data for a single patient could not be linked. The VICORI is a research platform that links patient-level records from the NHFA, with the NDRS, and the Office of National Statistics death registration. Detailed information on the VICORI linkage process has been previously published.^14^ More details are given in the *Supplementary Material*.

### Study population

All adults (≥18 years of age) with a first admission to the hospital for HF recorded in the NHFA from 1 January 2012 to 31 March 2018 (most recent NHFA data) were included; subsequent HF admissions were excluded. The NHFA data collection is nationally mandated and from 2012 contains high-quality data.^17^ We did not consider any first admission with HF recorded before 2012.

### Cohorts

We defined our cancer cohort as linked patients from the NCRD,^16^ diagnosed within 10 years before the HF admission with the most common tumour sites identified by ICD-10 coding: breast (C50 females only), prostate (C61 males only), colorectal (C18–C20), and trachea, bronchus, and lung cancer (C33–C34). We analysed the cancer patients together and stratified by tumour site, but data on the stage of cancer at the time of cancer diagnosis and cardiovascular risk factors were limited. The comparator population consisted of HF patients without a diagnosis of malignant cancer (i.e. not identified in the NCRD) in the 10 years prior to HF admission. Comparator patients for breast cancer were restricted to females, for prostate cancer were males, and for colorectal and lung cancer were all patients without cancer.

### Outcomes

Primary outcomes were (i) HF presentation (phenotype and in-hospital mortality), (ii) place of care (cardiology vs. non-cardiology vs. unknown ward care), (iii) HF medication prescribing, and (iv) post-discharge survival. Data completeness for left ventricular systolic dysfunction, as identified through echocardiography or other gold standard tests, is good and was used to identify heart failure with reduced ejection fraction (HFrEF). Heart failure severity was determined using the New York Heart Association (NYHA) Classification standard breathlessness score (1, least severe; 4, most severe including symptoms at rest/increase with physical activity).^18^

For patients with HFrEF discharged alive from hospital, HF management medications prescribed at discharge were obtained from the NHFA and included ACEi/ARBs, beta-blockers, loop diuretics, MRAs, and digoxin. Hospital discharge medications are only reported for patients with HFrEF because these medications are indicated for patients with HFrEF. Finally, for patients that did not die in the hospital, the date of death was obtained from the Office of National Statistics for post-discharge survival analyses. Patients were censored at the end of the study, 26 November 2018.

### Statistical analyses

Stata/SE 15.1 was used for all analyses. Propensity weighting ensures that the distribution of known confounders is the same across exposure groups.^19^ In this study, we reweighted the distribution of confounders in patients without cancer to that of patients with cancer to provide estimates of the average effects in a cancer population.^20^ Potential confounding factors were selected *a priori* from the NHFA and consisted of age at HF admission, sex, ethnicity (categorized as White, Black, South Asian, other, unknown), year of HF admission, and the following pre-existing diseases: ischaemic heart disease, valve disease, diabetes, and chronic obstructive pulmonary disease. For cancer patients, ethnicity was obtained from the NDRS if unavailable in the NHFA.

Propensity scores were calculated for the comparison of the overall cancer cohort to patients without cancer and separately for each cancer site in comparison to patients without cancer. We used propensity score weighting to estimate the percentage of patients with and without cancer that experienced each outcome and the difference with 95% confidence interval. Standardized differences in baseline characteristics at HF admission were examined before and after propensity weighting for each exposure comparison.

Flexible parametric survival models were used to examine post-hospital discharge survival. All survival analyses excluded patients who died in the hospital or in whom hospital discharge date was after the end of survival follow-up. A restricted cubic spline was used to model the baseline log cumulative hazard of mortality, with four degrees of freedom. Cancer status was included as a binary variable with an interaction with follow-up time to allow for non-proportional hazards.^21^ Crude survival plots were created for cancer and non-cancer populations prior to any adjustment for confounders. For adjusted survival plots, age at admission, calendar year of admission, sex, ethnicity, and pre-existing diseases were included as covariates in the survival model, with restricted cubic splines used for age and calendar year of admission. Adjusted post-discharge survival curves were obtained for each exposure comparison, standardizing to the covariate distribution of cancer patients.

Analyses of post-discharge non-cancer–related mortality was investigated by censoring deaths from any cancer, ICD-10 C00-C97, using underlying cause of death information obtained from the Office of National Statistics (via the NDRS). For patients without cancer at the time of HF diagnosis, we searched for any future linkage with the NDRS and recorded cause of death from the Office of National Statistics if available. For non-cancer patients with no linkage to the NDRS, cause of death information was not available, and we made an assumption that these patients did not die of cancer. We obtained non-cancer net survival estimates, which describe survival free from non-cancer–related mortality in a population where cancer deaths cannot apply.

We investigated effect modification by grouping cancer into four distinct groups: lung cancer, non-lung cancers (breast, prostate, or colorectal) with recent diagnosis (≤1 year), non-lung cancers with diagnosis >1 and ≤3 years before HF, and non-lung cancers with diagnosis >3 years before HF. For each group, we estimated the difference in discharge medication prescription and all-cause mortality compared with controls.

### Patient and public involvement

A group of patient representatives provided the study team with information on the experience of patients with cancer and heart disease and guided the key questions for the VICORI programme. The lead patient representatives attended the study management group meetings, provided guidance on study design and prioritization of research questions, and ensured study information and findings are disseminated, available, and accessible to patients and the public.

## Results

The HF cohort comprised 221 953 patients admitted to the hospital for HF including 12 867 (5.8%) patients with a prior cancer diagnosis (of breast, prostate, colorectal, or lung cancer) and 209 086 without a cancer diagnosis (*Table 1*). Mean age was 78.1 years (SD 12.6) and 53.7% of the patients were male. Most patients were White (54.9%) or of unknown ethnicity (39.1%). There was a high prevalence of pre-existing diseases including ischaemic heart disease (38.8%), diabetes (29.9%), valve disease (21.7%), and chronic obstructive pulmonary disease (16.2%). Approximately half of patients presented with HFrEF, and most of these hospitalized patients (72.8%) had NYHA Classification 3 or 4 (*Table 1*).

**Table 1:** Differences in baseline cohort characteristics before and after propensity weighting between patients with and without cancer who were admitted to the hospital for heart failure during 1 January 2012–31 March 2018

Baseline	Total	Before propensity weighting				After propensity weighting			
		Cancer		Difference	Standardized difference	Cancer		Difference	Standardized difference
		Yes	No			Yes	No		
Number of patients	221 953	12 867	209 086	—	—	12 867	209 086	—	—
Age (years), mean (SD)	78.1 (12.6)	80.8 (8.9)	77.9 (12.7)	2.8	−0.259	80.8	80.8	0.0	0.001
Sex, *n* (%)									
Male	119 241 (53.7%)	7923 (61.6%)	111 318 (53.2%)	8.4	0.169	61.6%	61.4%	0.2	0.003
Female	102 712 (46.3%)	4944 (38.4%)	97 768 (46.8%)	−8.4		38.4%	38.6%	−0.2	−0.003
Ethnicity, *n* (%)									
Unknown	86 852 (39.1%)	4769 (37.1%)	82 083 (39.3%)	−2.2	0.045	37.1%	37.1%	0.0	0.000
Black	3245 (1.5%)	176 (1.4%)	3069 (1.5%)	−0.1	0.008	1.4%	1.4%	0.0	0.001
Other	7806 (3.5%)	434 (3.4%)	7372 (3.5%)	−0.1	0.008	3.4%	3.4%	0.0	0.000
South Asian	2279 (1.0%)	58 (0.5%)	2221 (1.1%)	−0.6	0.071	0.5%	0.5%	0.0	0.000
White	121 771 (54.9%)	7430 (57.7%)	114 341 (54.7%)	3.0	0.062	57.7%	57.7%	0.0	0.000
**Pre-existing conditions**									
Ischaemic heart disease, *n* (%)									
No	125 801 (56.7%)	7409 (57.6%)	118 392 (56.6%)	1.0	0.019	57.6%	57.6%	0.0	0.000
Yes	86 183 (38.8%)	4917 (38.2%)	81 266 (38.9%)	−0.7	0.013	38.2%	38.2%	0.0	0.000
Unknown	9969 (4.5%)	541 (4.2%)	9428 (4.5%)	−0.3	0.015	4.2%	4.2%	0.0	0.000
Valve disease, *n* (%)									
No	162 473 (73.2%)	9536 (74.1%)	152 937 (73.1%)	1.0	0.022	74.1%	74.1%	0.0	0.000
Yes	48 270 (21.7%)	2742 (21.3%)	45 528 (21.8%)	−0.5	0.011	21.3%	21.3%	0.0	0.000
Unknown	11 210 (5.1%)	589 (4.6%)	10 621 (5.1%)	−0.5	0.023	4.6%	4.6%	0.0	0.000
Diabetes, *n* (%)									
No	148 740 (67.0%)	8967 (69.7%)	139 773 (66.8%)	2.9	0.061	69.7%	69.7%	0.0	0.000
Yes	66 419 (29.9%)	3531 (27.4%)	62 888 (30.1%)	−2.7	0.058	27.4%	27.5%	−0.1	0.000
Unknown	6794 (3.1%)	369 (2.9%)	6425 (3.1%)	−0.2	0.012	2.9%	2.9%	0.0	0.000
Chronic obstructive pulmonary disease, *n* (%)									
No	174 230 (78.5%)	9954 (77.4%)	164 276 (78.6%)	−1.2	0.029	77.4%	77.3%	0.1	0.000
Yes	36 013 (16.2%)	2306 (17.9%)	33 707 (16.1%)	1.8	0.048	17.9%	17.9%	0.0	0.000
Unknown	11 710 (5.3%)	607 (4.7%)	11 103 (5.3%)	−0.6	0.027	4.7%	4.7%	0.0	0.000
**HF presentation**									
HFrEF, *n* (%)									
No	97 927 (44.1%)	5838 (45.4%)	92 089 (44.0%)	1.4	0.027	45.4%	44.7%	0.7	0.013
Yes	119 446 (53.8%)	6750 (52.5%)	112 696 (53.9%)	−1.4	0.029	52.5%	53.2%	−0.7	−0.016
Unknown	4580 (2.1%)	279 (2.2%)	4301 (2.1%)	0.1	0.008	2.2%	2.0%	0.2	0.010
NYHA Classification, *n* (%)									
1	13 904 (6.3%)	763 (5.9%)	13 141 (6.3%)	−0.4	0.015	5.9%	6.2%	−0.3	−0.012
2	31 697 (14.3%)	1753 (13.6%)	29 944 (14.3%)	−0.7	0.020	13.6%	14.1%	−0.5	−0.014
3	90 471 (40.8%)	5263 (40.9%)	85 208 (40.8%)	0.1	0.003	40.9%	41.0%	−0.1	−0.002
4	71 043 (32.0%)	4273 (33.2%)	66 770 (31.9%)	1.3	0.027	33.2%	32.2%	1.0	0.022
Unknown	14 838 (6.7%)	815 (6.3%)	14 023 (6.7%)	−0.4	0.015	6.3%	6.5%	−0.2	−0.007

HFrEF, failure with reduced ejection fraction; NYHA, New York Heart Association. Age is listed in years as mean (SD). All other information is number (%).

Propensity score includes the variables: age at HF admission, sex, ethnicity year of HF admission, ischaemic heart disease, valve disease, diabetes, and chronic obstructive pulmonary disease.

Differences in baseline characteristics between patients with and without cancer were eliminated after propensity score weighting (all standardized differences <0.005, *Table 1*), with propensity score distributions exhibiting satisfactory overlap between HF patients with and without cancer. Similarly, baseline differences between each tumour site and the corresponding patients without cancer were eliminated after propensity score weighting (all standardized differences <0.001, Supplementary material online, *Table S1*).

Amongst cancer patients, there were 3216 (25.0%) breast, 5118 (39.8%) prostate, 3199 (24.9%) colorectal, and 1334 (10.4%) lung cancer patients. Nearly half (47.1%) were missing cancer stages with lung cancer patients having a higher proportion of advanced disease than other tumour sites (see Supplementary material online, *Table S2*). The cancer stage distribution varied greatly between the different cancer sites.

### Heart failure presentation and in-hospital outcomes

There were minimal differences in HF phenotype and severity (NYHA Classification), between patients with and without prior cancer, except in lung cancer patients where a lower percentage presented with HFrEF compared with patients without cancer [−4.3% age point difference (ppd) (95% CI −7.0, −1.6) after adjustment] (*Table 2*). A lower percentage of cancer patients were cared for in a cardiology ward compared with patients without cancer [−2.4 ppd (95% CI −3.3, −1.6) after adjustment; *Table 2*]. This was most pronounced in lung cancer patients where only 33.7% received care in a cardiology ward compared with 43.7% of patients without cancer [−10.1 ppd (95% CI −12.6, −7.5) after adjustment]. In-hospital mortality was 5.9% in cancer patients compared with 5.0% in patients without cancer [0.7 ppd (95% CI 0.3, 1.1) after adjustment]. The difference was highest between patients with lung cancer (7.0%) vs. without [4.6%, 2.4 ppd (95% CI 1.0, 3.8) after adjustment] (*Table 2*).

**Table 2:** Propensity-weighted difference (95% confidence interval) in primary outcomes and discharge medication for patients with heart failure admission by cancer diagnosis and tumour site

Outcome		Propensity-weighted														
	Total	Cancer			Tumour site											
		Yes	No	Diff (95% CI)		Breast cancer			Prostate cancer			Colorectal cancer		Lung cancer		
					Yes	No	Diff (95% CI)	Yes	No	Diff (95% CI)	Yes	No	Diff (95% CI)	Yes	No	Diff (95% CI)
Number of patients	221 953	12 867	209 086		3216	97 768		5118	111 318		3199	209 086		1334	209 086	
**HF hospital admission**																
Main place of care																
Other	123 521 (55.7%)	59.7%	57.2%	2.5 (1.6, 3.3)	62.4%	61.5%	0.9 (−0.8, 2.6)	56.5%	54.2%	2.3 (0.9, 3.7)	59.4%	58.1%	1.4 (−0.4, 3.1)	66.0%	55.9%	10.1 (7.5, 12.6)
Cardiology	97 592 (44.0%)	40.0%	42.4%	−2.4 (−3.3, −1.6)	37.2%	38.1%	−0.9 (−2.6, 0.8)	43.2%	45.4%	−2.2 (−3.6, −0.8)	40.2%	41.6%	−1.3 (−3.0, 0.4)	33.7%	43.7%	−10.1 (−12.6, −7.5)
Unknown	840 (0.4%)	0.3%	0.4%	0.0 (−0.1, 0.1)	0.4%	0.4%	0.0 (−0.2, 0.2)	0.3%	0.4%	−0.1 (−0.2, 0.1)	0.3%	0.4%	0.0 (−0.2, 0.2)	0.4%	0.4%	0.0 (−0.3, 0.3)
Died in the hospital																
No	211 249 (95.2%)	94.2%	94.9%	−0.7 (−1.1, −0.3)	94.7%	95.2%	−0.5 (−1.3, 0.3)	94.1%	94.6%	−0.5 (−1.2, 0.2)	94.2%	94.6%	−0.4 (−1.2, 0.4)	93.0%	95.4%	−2.4 (−3.8, −1.0)
Yes	10 704 (4.8%)	5.9%	5.0%	0.7 (0.3, 1.1)	5.3%	4.8%	0.5 (−0.3, 1.3)	5.9%	5.4%	0.5 (−0.2, 1.2)	5.8%	5.4%	0.4 (−0.4, 1.2)	7.0%	4.6%	2.4 (1.0, 3.8)
**HF presentation**																
HFrEF, *n* (%)																
No	97 927 (44.1%)	45.4%	44.7%	0.6 (−0.3, 1.5)	53.3%	53.9%	−0.5 (−2.3, 1.2)	39.9%	38.8%	1.1 (−0.3, 2.5)	45.2%	45.2%	0.0 (−1.8, 1.7)	47.6%	43.7%	3.9 (1.2, 6.6)
Yes	119 446 (53.8%)	52.5%	53.2%	−0.8 (−1.7, 0.1)	44.1%	43.9%	0.1 (−1.6, 1.9)	58.0%	59.2%	−1.2 (−2.6, 0.1)	53.0%	52.8%	0.3 (−1.5, 2.0)	50.0%	54.3%	−4.3 (−7.0, −1.6)
Unknown	4580 (2.1%)	2.2%	2.0%	0.1 (−0.1, 0.4)	2.6%	2.2%	0.4 (−0.2, 0.9)	2.1%	2.0%	0.1 (−0.3, 0.5)	1.8%	2.0%	−0.3 (−0.7, 0.2)	2.4%	1.9%	0.5 (−0.4, 1.3)
NYHA Classification, *n* (%)																
1	13 904 (6.3%)	5.9%	6.2%	−0.3 (−0.7, 0.1)	5.5%	6.2%	−0.7 (−1.5, 0.1)	6.5%	6.4%	0.2 (−0.5, 0.9)	5.9%	6.2%	−0.3 (−1.1, 0.5)	4.7%	5.6%	−0.9 (−2.1, 0.2)
2	31 697 (14.3%)	13.6%	14.1%	−0.5 (−1.1, 0.1)	13.4%	14.3%	−0.9 (−2.1, 0.3)	14.2%	14.0%	0.2 (−0.8, 1.2)	13.7%	14.0%	−0.4 (−1.6, 0.8)	11.8%	13.7%	−1.9 (−3.6, −0.1)
3	90 471 (40.8%)	40.9%	41.0%	−0.1 (−1.0, 0.8)	40.9%	40.6%	0.3 (−1.4, 2.0)	41.3%	41.3%	0.0 (−1.4, 1.4)	39.5%	41.0%	−1.4 (−3.1, 0.3)	42.7%	41.6%	1.1 (−1.5, 3.8)
4	71 043 (32.0%)	33.2%	32.2%	1.0 (0.2, 1.9)	33.6%	32.3%	1.3 (−0.3, 3.0)	31.9%	31.9%	0.0 (−1.3, 1.3)	34.4%	32.3%	2.0 (0.4, 3.7)	34.5%	33.0%	1.5 (−1.1, 4.0)
Unknown	14 838 (6.7%)	6.3%	6.5%	−0.2 (−0.6, 0.3)	6.6%	6.7%	−0.1 (−1.0, 0.8)	6.1%	6.4%	−0.4 (−1.0, 0.3)	6.5%	6.5%	0.0 (−0.8, 0.9)	6.2%	6.0%	0.2 (−1.1, 1.5)
**Discharge medication** ^a^																
Number of patients with HFrEF and did not die in the hospital	114 001	6385	107 616	—	1355	40 913	—	2794	66 703	—	1604	107 616	—	632	107 616	—
ACEi/ARB																
No	28 341 (24.9%)	28.4%	26.7%	1.7 (0.6, 2.9)	25.5%	26.3%	−0.8 (−3.2, 1.6)	28.8%	27.3%	1.6 (−0.1, 3.3)	30.0%	27.7%	2.3 (0.1, 4.6)	29.1%	25.1%	4.1 (0.5, 7.6)
Yes	78 037 (68.5%)	64.1%	66.2%	−2.1 (−3.3, −0.9)	67.6%	66.9%	0.7 (−1.8, 3.2)	63.8%	65.4%	−1.7 (−3.5, 0.2)	62.3%	65.0%	−2.7 (−5.1, −0.3)	62.5%	68.4%	−5.9 (−9.7, −2.1)
Unknown/NA	7623 (6.7%)	7.5%	7.1%	0.4 (−0.3, 1.1)	6.9%	6.8%	0.1 (−1.3, 1.5)	7.4%	7.3%	0.1 (−0.9, 1.1)	7.7%	7.3%	0.4 (−0.9, 1.7)	8.4%	6.6%	1.8 (−0.3, 4.0)
Beta-blocker																
No	22 044 (19.3%)	21.0%	20.6%	0.4 (−0.7, 1.4)	19.9%	20.3%	−0.4 (−2.5, 1.8)	20.8%	21.1%	−0.2 (−1.8, 1.3)	20.9%	21.0%	−0.2 (−2.2, 1.9)	24.1%	20.9%	3.1 (−0.2, 6.5)
Yes	84 052 (73.7%)	71.5%	72.0%	−0.6 (−1.7, 0.6)	73.3%	72.6%	0.7 (−1.7, 3.1)	71.7%	71.3%	0.4 (−1.3, 2.1)	71.1%	71.4%	−0.3 (−2.5, 2.0)	67.4%	72.1%	−4.7 (−8.4, −1.1)
Unknown/NA	7905 (6.9%)	7.6%	7.4%	0.2 (−0.5, 0.9)	6.8%	7.1%	−0.3 (−1.7, 1.1)	7.5%	7.6%	−0.2 (−1.2, 0.8)	8.0%	7.6%	0.4 (−0.9, 1.8)	8.5%	6.9%	1.6 (−0.5, 3.8)
Loop diuretic																
No	12 507 (11.0%)	10.9%	10.2%	0.6 (−0.2, 1.4)	10.7%	10.8%	−0.1 (−1.8, 1.5)	10.4%	10.0%	0.4 (−0.8, 1.6)	10.8%	10.0%	0.8 (−0.8, 2.3)	13.3%	10.3%	2.9 (0.3, 5.6)
Yes	93 848 (82.3%)	82.4%	83.0%	−0.6 (−1.6, 0.3)	83.0%	82.7%	0.2 (−1.8, 2.3)	82.9%	83.0%	−0.2 (−1.6, 1.3)	82.5%	83.2%	−0.7 (−2.5, 1.2)	79.0%	83.2%	−4.3 (−7.4, −1.1)
Unknown/NA	7646 (6.7%)	6.8%	6.7%	0.0 (−0.6, 0.6)	6.3%	6.4%	−0.1 (−1.4, 1.2)	6.7%	7.0%	−0.2 (−1.2, 0.7)	6.7%	6.8%	−0.1 (−1.3, 1.1)	7.8%	6.4%	1.3 (−0.8, 3.4)
MRA																
No	51 823 (45.5%)	48.1%	46.9%	1.2 (−0.1, 2.5)	48.0%	48.3%	−0.3 (−3.0, 2.4)	47.0%	46.6%	0.4 (−1.5, 2.3)	49.0%	47.6%	1.4 (−1.1, 3.8)	50.6%	45.4%	5.2 (1.3, 9.2)
Yes	45 363 (39.8%)	36.1%	37.8%	−1.7 (−2.9, −0.5)	36.5%	36.0%	0.5 (−2.1, 3.1)	37.6%	38.1%	−0.5 (−2.4, 1.3)	34.9%	36.8%	−1.9 (−4.3, 0.4)	32.3%	39.8%	−7.6 (−11.2, −3.9)
Unknown/NA	16 815 (14.7%)	15.8%	15.3%	0.5 (−0.4, 1.4)	15.5%	15.7%	−0.2 (−2.2, 1.7)	15.4%	15.3%	0.2 (−1.2, 1.5)	16.1%	15.6%	0.6 (−1.3, 2.4)	17.1%	14.8%	2.3 (−0.6, 5.3)
Digoxin																
No	70 564 (61.9%)	61.1%	61.4%	−0.4 (−1.6, 0.9)	60.6%	60.8%	−0.2 (−2.8, 2.5)	61.8%	62.1%	−0.3 (−2.1, 1.6)	61.0%	61.6%	−0.6 (−3.0, 1.8)	59.0%	60.8%	−1.8 (−5.6, 2.1)
Yes	17 998 (15.8%)	16.3%	16.1%	0.2 (−0.8, 1.1)	18.5%	17.2%	1.3 (−0.8, 3.4)	15.1%	15.1%	0.0 (−1.3, 1.4)	16.7%	15.9%	0.8 (−1.0, 2.6)	16.0%	16.9%	−0.9 (−3.8, 2.0)
Unknown/NA	25 439 (22.3%)	22.6%	22.4%	0.2 (−0.9, 1.3)	21.0%	22.0%	−1.1 (−3.3, 1.1)	23.0%	22.8%	0.3 (−1.3, 1.9)	22.3%	22.5%	−0.2 (−2.3, 1.9)	25.0%	22.4%	2.6 (−0.8, 6.0)

HF, heart failure; HFrEF, heart failure with reduced ejection fraction; NYHA, New York Heart Association; ACEi/ARB, angiotensin-converting enzyme inhibitors or angiotensin receptor antagonists; MRA, mineralocorticoid receptor antagonists; Diff, difference. Lung cancer includes trachea, bronchus, and lung cancers. The no breast cancer group includes females only, whilst the no prostate cancer group includes males only.

Propensity score includes the variables: age at HF admission, sex, ethnicity year of HF admission, ischaemic heart disease, valve disease, diabetes, and chronic obstructive pulmonary disease.

Discharge medication was evaluated for patients who did not die in the hospital and had left ventricular systolic dysfunction.

### Discharge medications

Of the 114 001 HF patients who had HFrEF and were discharged alive, 64.1% of the cancer patients received ACEi/ARB on hospital discharge compared with 66.2% of patients without cancer [−2.1 ppd (95% CI −3.3, −0.9) after adjustment; *Table 2*, Supplementary material online, *Figure S1*], driven primarily by lower prescribing in the lung cancer population [62.5%; −5.9 ppd (95% CI −9.7, −2.1) after adjustment]. The percentage of patients with and without cancer that were prescribed other HF management medications was comparable (≤2 ppd after adjustment) except for lung cancer patients where fewer patients received MRA [−7.6 ppd (95% CI −11.2, −3.9) after propensity score adjustment], beta-blockers [−4.7 ppd (95% CI −8.4, −1–1)], and loop diuretics [−4.3 ppd (95% CI −7.4, −1.1)] compared with patients without lung cancer. Amongst HF patients with non-lung cancer, there was no clear effect of time since cancer diagnosis on the likelihood of being prescribed HF discharge medication (see Supplementary material online, *Figure S2*).

### Post-discharge survival

**Figure 1:**
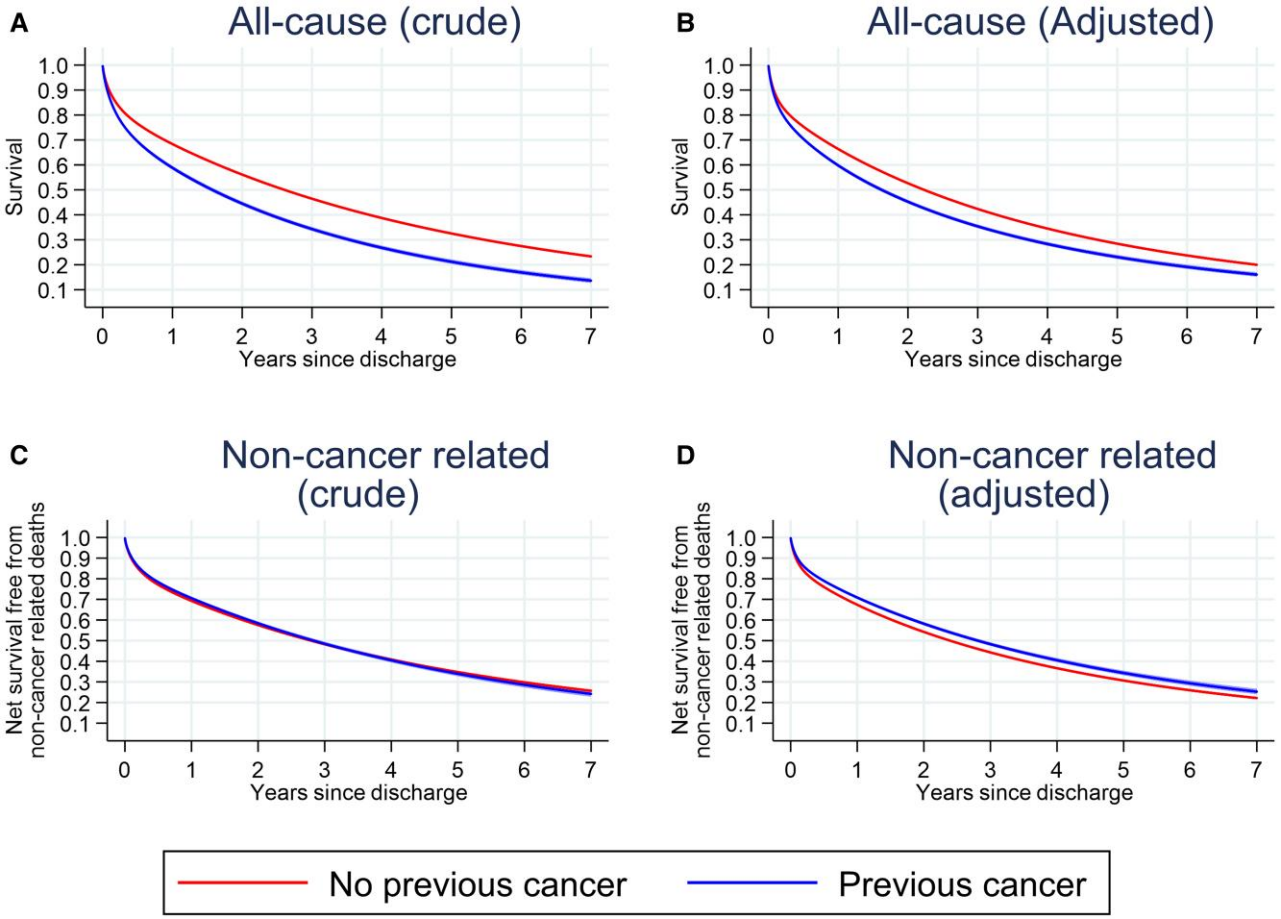
All-cause survival, post-hospital discharge, for heart failure by cancer diagnosis. Survival rates after hospital discharge for heart failure by cancer diagnosis with differing levels of adjustment. (*A*) Crude and (*B*) adjusted non-cancer–related net survival; (*C*) crude and (*D*) adjusted non-cancer related. Adjusted for age at admission, year of admission, sex, ethnicity (White, Black, South Asian, other, unknown), New York Heart Association class, and the following pre-existing diseases: valve disease, ischaemic heart disease, diabetes, and chronic obstructive pulmonary disease. *n* = 211 224 patients discharged alive and with available post-discharge follow-up.

**Figure 2:**
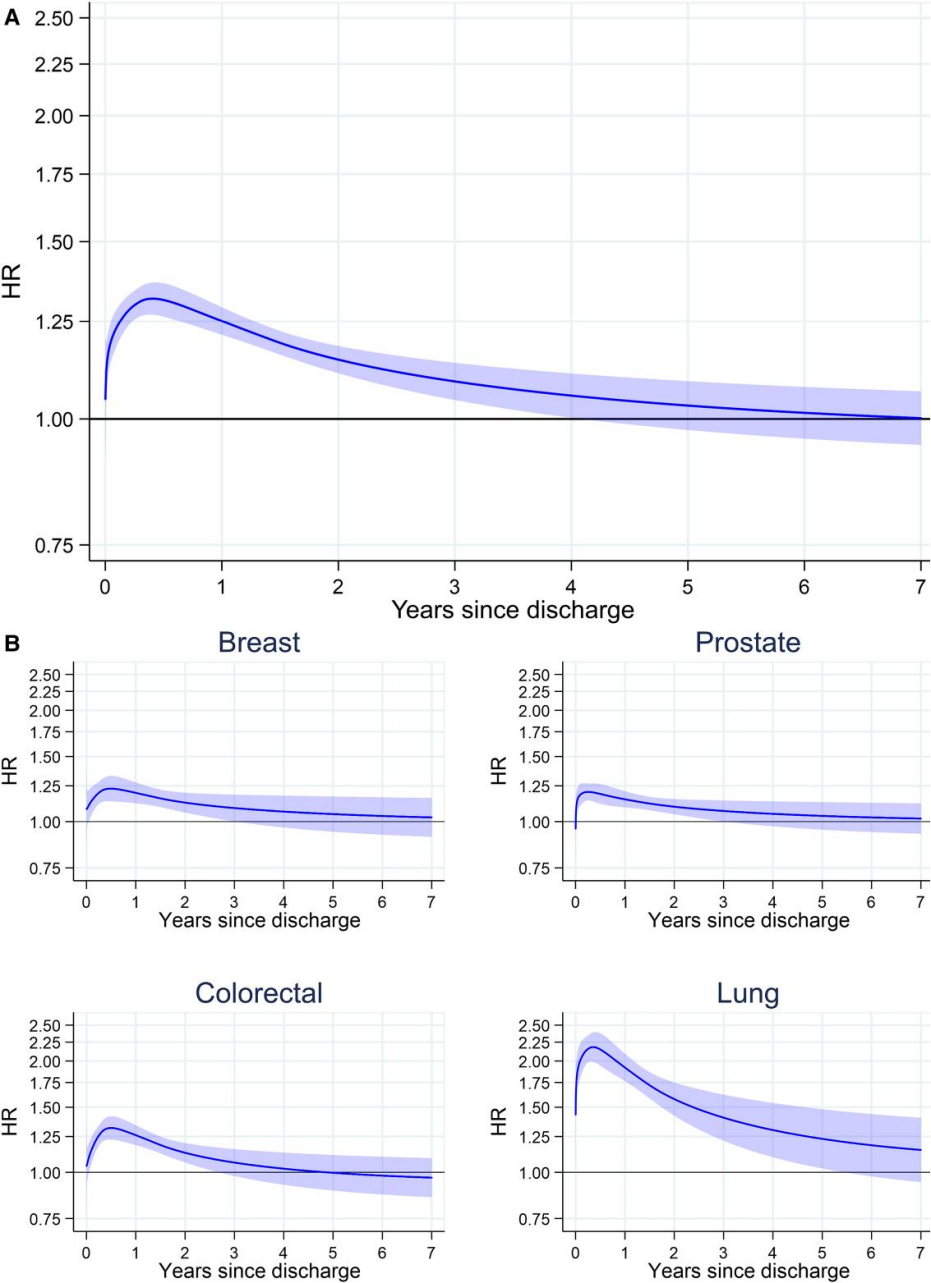
Hazard ratios for post-discharge mortality compared with no previous cancer. Marginal hazard ratios for post-discharge mortality comparing cancer to no previous cancer. (*A*) Marginal hazard ratio of mortality in all cancer patients relative to patients without cancer. (*B*) Marginal hazard ratio of mortality in breast cancer relative to women without cancer, prostate cancer relative to men without cancer, both colorectal and lung cancer are relative to patients without cancer. Adjusted for age at admission, year of admission, sex, ethnicity (White, Black, South Asian, other, unknown), New York Heart Association class, and the following pre-existing diseases: valve disease, ischaemic heart disease, diabetes, and chronic obstructive pulmonary disease. Lung cancer includes trachea, bronchus, and lung cancers. *n* = 211 224 patient discharged alive and with available post-discharge follow-up.

Survival post-acute HF discharge was estimated in 211 224 patients discharged alive with available follow-up. There were 117 533 post-discharge deaths during a mean follow-up time of 2.0 years (SD 1.7, range 0.0–6.9). Overall survival after an acute heart failure admission was low, regardless of prior cancer status. Median post-discharge survival was 2.5 years (95% CI 2.4, 2.5), 1.6 years (95% CI 1.5, 1.6) for patients with cancer, and 2.6 years (95% CI 2.6, 2.6) for patients without cancer (*Figure 1A*). This difference was attenuated but remained after adjusting for baseline characteristics (*Figure 1B*). A significantly increased rate of death for cancer patients was present in the first few years after hospital discharge and remained elevated in lung cancer patients up to 5 years after discharge (*Figure 2*). Despite this, most of the 7569 post-discharge deaths in cancer patients had non-cancer–related underlying causes with 3261 (41%) from diseases of the circulatory system and a further 920 (12%) from respiratory diseases. This compared with 2568 (32%) deaths with malignant neoplasm as the underlying cause (*Table 3*). Patients with and without cancer had similar non-cancer–related net survival, which was extremely poor for both groups of patients (*Figures 1C* and *1D*).

**Table 3:** Distribution of underlying causes of death after hospital discharge for HF in patients with prior cancer

ICD-10		Description	*n* (%)
A	A00-B99	Certain infectious and parasitic diseases	68 (0.9)
B			5 (0.1)
C	C00-C97	Malignant neoplasms	2568 (32.3)
D	D00-D48	Other neoplasms	37 (0.5)
	D50-D89	Diseases of the blood and blood-forming organs and certain disorders involving the immune mechanism	8 (0.1)
E	E00-E89	Endocrine, nutritional, and metabolic diseases	128 (1.6)
F	F01-F99	Mental, behavioural, and neurodevelopmental disorders	206 (2.6)
G	G00-G99	Diseases of the nervous system	90 (1.1)
H	H00-H59	Diseases of the eye and adnexa	0 (0.0)
	H60-H95	Diseases of the ear and mastoid process	0 (0.0)
I	I00-I99	Diseases of the circulatory system	3261 (41.0)
	I00-I02	− of which acute rheumatic fever	0 (0.0)
	I05-I09	− of which chronic rheumatic heart diseases	32 (0.4)
	I10-I16	− of which hypertensive diseases	138 (1.7)
	I20-I25	− of which ischaemic heart diseases	1681 (21.1)
	I26-I28	− of which pulmonary heart disease and diseases of pulmonary circulation	51 (0.6)
	I30-I49	− of which other forms of heart disease	635 (8.0)
	I50–15A	− of which heart failure	447 (5.6)
	I60-I69	− of which cerebrovascular diseases	206 (2.6)
	I70-I79	− of which diseases of arteries, arterioles, and capillaries	63 (0.8)
	I80-I89	− of which diseases of veins, lymphatic vessels, and lymph nodes, not elsewhere classified	8 (0.1)
	I95-I99	− of which other and unspecified disorders of the circulatory system	0 (0.0)
J	J00-J99	Diseases of the respiratory system	920 (11.6)
K	K00-K95	Diseases of the digestive system	206 (2.6)
L	L00-L99	Diseases of the skin and subcutaneous tissue	46 (0.6)
M	M00-M99	Diseases of the musculoskeletal system and connective tissue	46 (0.6)
N	N00-N99	Diseases of the genitourinary system	148 (1.9)
O	O00-O99	Pregnancy, childbirth, and the puerperium	0 (0.0)
P	P00-P96	Certain conditions originating in the perinatal period	0 (0.0)
Q	Q00-Q99	Congenital malformations, deformations, and chromosomal abnormalities	9 (0.1)
R	R00-R99	Symptoms, signs, and abnormal clinical and laboratory findings, not elsewhere classified	62 (0.8)
S	S00-T88	Injury, poisoning, and certain other consequences of external causes	0 (0.0)
T			0 (0.0)
V	V00-Y99	External causes of morbidity	3 (0.0)
Y			3 (0.0)
Z	Z00-Z99	Factors influencing health status and contact with health services	0 (0.0)
Multiple underlying causes of death recorded (usually indicating deaths due to accidents, poisonings, and violence)			102 (1.3)
Missing			40 (0.5)
**Total deaths**			**7948 (100.0)**

**Figure 3:**
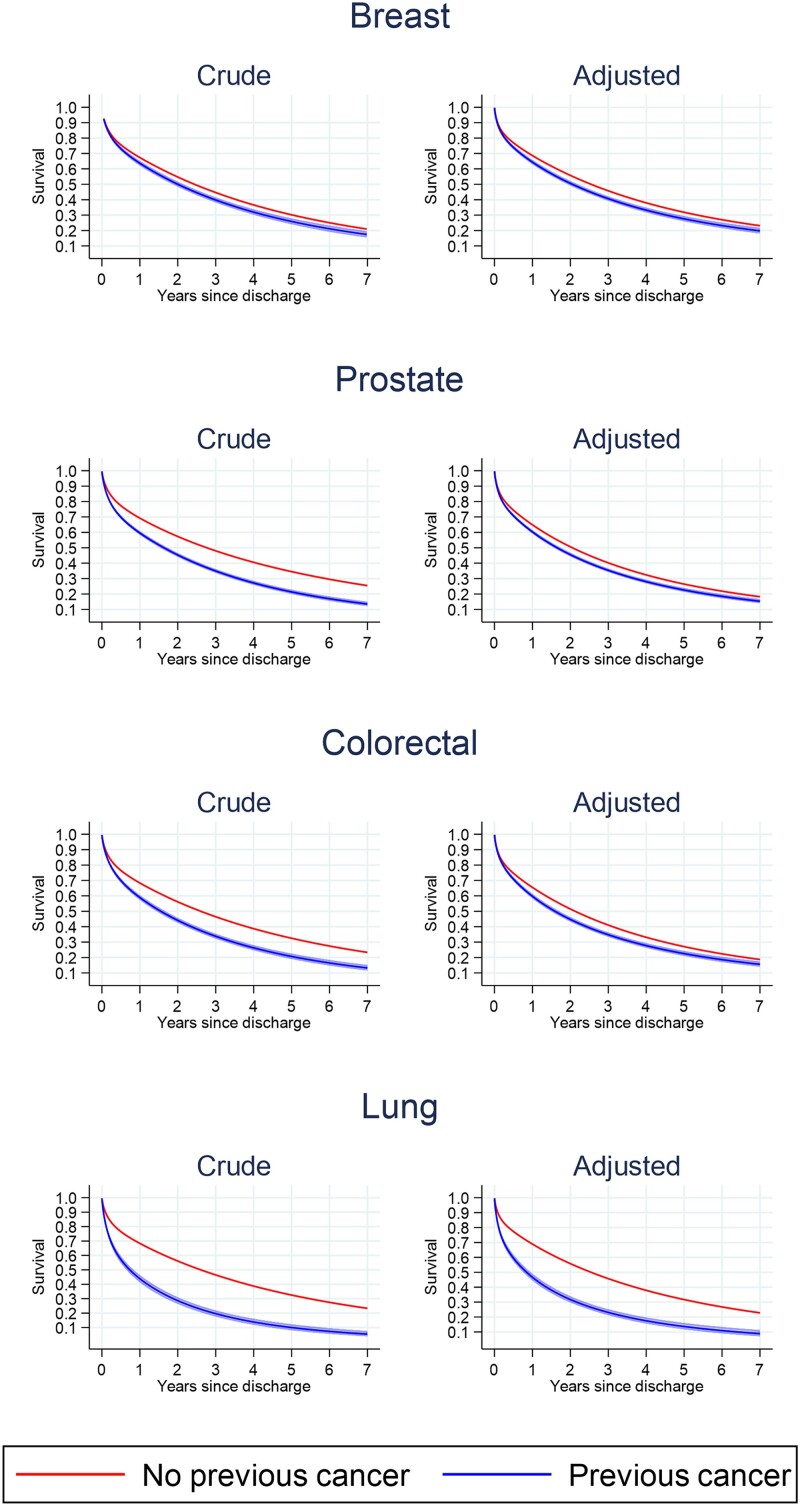
All-cause survival post-hospital discharge for heart failure by tumour site. Crude and adjusted all-cause survival post-hospital discharge for each tumour site compared with patients without cancer. Each tumour site compared with patients without cancer. Crude and adjusted for age at admission, year of admission, sex, ethnicity (White, Black, South Asian, other, unknown), New York Class Association, and the following pre-existing diseases: valve disease, ischaemic heart disease, diabetes, and chronic obstructive pulmonary disease. The no breast cancer group includes females only, whilst the no prostate cancer group includes males only. Lung cancer includes trachea, bronchus, and lung cancers. *n* = 211 224 patients discharged alive and with available post-discharge follow-up.

Lung cancer patients had the worst median survival post-acute HF discharge. Median survival was 2.0 years (95% CI 1.9, 2.1) for women with breast cancer, 1.6 years (95% CI 1.5, 1.7) for men with prostate cancer, 1.5 years (95% CI 1.4, 1.6) for patients with colorectal cancer, 0.8 years (95% CI 0.7, 0.9) for patients with lung cancer, and 2.6 years (95% CI 2.6, 2.6) for patients without cancer (2.4 years for women and 2.7 years for men) (*Figure 3*). After adjustment for confounders, survival differences remained between prior cancer and non-cancer patients, though to a lesser degree in comparisons with non-lung (breast, prostate, and colorectal) cancer cohorts. Amongst non-lung cancer patients admitted with acute HF, prognosis was slightly poorer in patients with a recent cancer diagnosis, whilst for patients diagnosed more than 3 years ago, prognosis was similar to patients without cancer (see Supplementary material online, *Figure S3*).

## Discussion

We identified a large, diverse, nationally representative cohort of 12 867 cancer survivors and 209 086 patients without cancer admitted to the hospital with HF. In this cohort, we found, firstly, survival following hospital discharge for HF was very poor for prior cancer patients with only 23% remaining alive at 5 years. Secondly, whilst survivors of prior cancer had worse survival following HF compared with patients without previous cancer, mortality in cancer patients with HF was driven primarily by non-cancer causes. As a result, and particularly in non-lung cancer patients, differences in adjusted survival between cancer and non-cancer patients following HF discharge were relatively small. Thirdly, fewer survivors of prior cancer admitted to the hospital with HF were managed by cardiology specialists compared with similar patients without cancer, and finally, survivors of prior cancer presenting with HFrEF were less likely to receive guideline-based therapies,^12^ particularly ACEi/ARB, compared with similar patients without cancer. This disparity was most evident for lung cancer survivors.

Cardiovascular co-morbidities in cancer patients may arise as a direct consequence of complications of cancer or cancer treatment, shared cancer–cardiovascular risk factors, or simply as coincidental diseases.^2,4–6^ As cancer treatment and outcomes improve, optimal management of cardiovascular co-morbidities is increasingly important in further improving survival.^22^ This study supports this new cardio-oncological paradigm. It is perhaps not surprising that the combination of previous cancer and an admission to the hospital with acute heart failure in this study carried a particularly dire prognosis.^23^ However, importantly, from the adjusted survival analysis presented, it appears that mortality in the cancer population is driven primarily by non-cancer causes of death. The partial exception is lung cancer patients and to a lesser extent those with a recent cancer diagnosis (where the cancer prognosis is worse). These findings suggest that in cancer patients presenting with a heart failure admission, improving heart failure care and management of co-morbidities in particular has the potential for improving survival.

Given this, our findings of potential deficits in specialist hospital care and evidence-based management suggest there may be opportunities to improve outcomes in cancer patients presenting with acute heart failure. Specifically, we have shown cancer patients with HFrEF were less likely than patients without cancer to be prescribed ACEi/ARB with the largest effect seen in lung cancer patients in whom a deficit of MRA and beta-blocker prescribing was also noted. This supports other research showing that HF management therapies are under-prescribed for cancer patients.^24^ For example, a study by Ohtani *et al.* found that only 51.9% of cancer patients who developed anthracycline-induced cardiotoxicity received HF management therapy including renin–angiotensin inhibitor and/or beta-blocker therapy.^25^ Another study found that only 48% of patients that experienced cardiotoxicity commenced on beta-blocker and/or ACEi therapy.^26^ In some cases, under-prescription may be appropriate due to a contraindication, for example, during the terminal phase of cancer care or where oral treatment is limited. However, it is well established that HF medications improve symptoms and reduce HF admissions as well as improve prognosis.^27–29^ There is therefore a strong rationale for optimizing treatments, even in non-curable cancers. Further research will be needed to determine to what extent the treatment differences demonstrated are a reflection of appropriate clinical management of patients with a poor cancer prognosis and whether improving treatment in this group has the potential to improve outcomes.

One of the primary reasons reported for underutilization of HF management therapy in cancer patients following hospital admission includes the absence of formal cardiology referral.^30^ Patients with HF looked after by an appropriate specialist multidisciplinary team receive significantly more guideline-recommended HF management therapy and have better outcomes.^10^ In a previous study, cardiology consultation in cancer patients has also been associated with a significantly higher frequency of HF management therapy prescription [100% vs. 52% for ACEi/ARB (*P* < 0.0001); 94% vs. 41% for beta-blocker (*P* < 0.0001)].^31^ Our study confirms that a lower percentage of cancer patients admitted with acute HF were seen in a cardiology ward. Again, the largest difference was seen in lung cancer patients. It is likely that patients whose main place of care is a non-cardiology ward do not receive a consultation with a cardiologist, which likely contributes to the suboptimal management of HF. These findings suggest that increased access to specialist cardiology or cardio-oncological care may improve treatment in cancer patients with HF.

### Limitations

The study has a number of limitations. The heart failure audit is limited to patients with an acute heart failure admission. This analysis cannot be extended to heart failure patients managed in primary care or an ambulatory setting. Propensity matching was limited to potentially relevant confounders reliably recorded in the audit data. Prescription of HF management medication at the point discharge was used as a proxy for continuing treatment. We did not have information on maintenance of medication at admission or for the duration of follow-up. The data analysed are retrospective, and therefore, we are at present unable to investigate newer heart failure medications such as angiotensin receptor–neprilysin inhibitor and sodium–glucose transporter 2 inhibitor therapies. Likewise, we are not able to include analysis of the timing and nature of cancer treatments. This will be an important future analysis. The NHFA does not include absolute values of ejection fraction, and so the field for left ventricular systolic dysfunction was used as a surrogate for HFrEF. Natriuretic peptide data are poorly completed with only 8% coverage in the audit and were therefore not used in this analysis. Cause of death information was only available for cancer patients from linked mortality data; thus, for the cause-specific analysis, it was assumed that cancer deaths were negligible in the controls. This was felt to be a safe assumption as the whole registry was searched for cancer diagnoses after HF presentation and death certificate cancer deaths are included in the registry. However, this meant that we could only compare non-cancer–related mortality between cases and controls, and we were unable to compare HF-specific mortality. Primary cause of death information may also be inaccurate in co-morbid populations, which may partly explain the apparent higher non-cancer–related survival in cancer patients (*Figure 1D*). Further, whereas tumour stage is now well recorded overall in the NCRD, tumour stage was not well-recorded in these patients with HF (53% missing) due to many cancers being diagnosed before 2012 where staging completeness was markedly lower. Therefore, we decided not to include tumour stage within the analyses. Cardiology ward care, as recorded in the audit, is used here as a surrogate for specialist care. We did not have information on prior cardiovascular risk factors or post-discharge care provided by HF community nurse specialists. We also did not have information about post-discharge quality of life. Finally, as for all observational studies, there is a risk of residual confounding limiting causal inferences.

## Conclusions

Survival of cancer patients presenting with acute HF is very poor and is driven primarily by non-cancer causes (i.e. HF). Cancer patients with acute HF are less likely to be managed by a cardiology specialist and are less likely to receive evidence-based treatments. This is particularly true for patients with a poorer cancer prognosis. More research will be needed to determine if these treatment differences are a reflection of appropriate prognosis-guided clinical management or if optimal HF management guided by a cardio-oncology specialist multidisciplinary team has any potential to improve outcomes in cancer patients.

## Supplementary Material

zuad020_Supplementary_Data

## Data Availability

Data cannot be shared for ethical/privacy reasons.
