# Supplementary material for: Acute heart failure presentation, management, and outcomes in cancer patients: a national longitudinal study
Source: Eur Heart J Acute Cardiovasc Care. 2023 Mar 8;12(5):315–27. doi: 10.1093/ehjacc/zuad020 (PMC10156472; doi:10.1093/ehjacc/zuad020)
Supplement: zuad020_Supplementary_Data [file zuad020_supplementary_data.docx]

**Acute heart failure presentation, management and outcomes in cancer patients: a real-world national longitudinal study**

**Supplement**

Contents

[**Data description** 3](#_Toc117860816)

[**Supplementary Table 1.** Propensity-weighted standardised differences in baseline cohort characteristics for patients with heart failure admission by tumour site for variables included in propensity scores 4](#_Toc117860817)

[**Supplementary Table 2.** Stage at cancer diagnosis by cancer site. 5](#_Toc117860818)

[**Supplementary Figure 1:** Percentage point differences in receiving HF discharge medication between patients with and without cancer who had HFrEF and were discharged alive (N = 114,001). 6](#_Toc117860819)

[**Supplementary Figure 2:** Percentage point differences in receiving HF discharge medication between patients with different cancer characteristics and patients without cancer who had HFrEF and were discharged alive (N = 114,001). Patients with cancer split by lung cancer, non-lung cancer diagnosed ≤1 year, non-lung cancer diagnosed >1 and ≤3 years, and non-lung cancer diagnosed >3 years before HF hospital admission. 7](#_Toc117860820)

[**Supplementary Figure 3:** Adjusted all-cause survival by lung cancer patients, non-lung cancer patients ≤1 year from diagnosis, non-lung cancer patients >1 and ≤3 years from diagnosis and non-lung cancer patients >3 years from diagnosis, compared to patients without cancer 8](#_Toc117860821)

# **Data description**

The National Heart Failure Audit (NHFA) is commissioned, as part of the ‘National Cardiac Audit Programme’ by the Healthcare Quality Improvement Partnership, which uses data provided by patients and collected by the NHS for patient care and support.

The cancer registry data are collated, maintained and quality assured by the National Disease Registration Service, part of NHS Digital.

# **Supplementary Table 1.** Propensity-weighted standardised differences in baseline cohort characteristics for patients with heart failure admission by tumour site for variables included in propensity scores

| **Baseline** | **Propensity-weighted** | | | | | | | | | | | |
| --- | --- | --- | --- | --- | --- | --- | --- | --- | --- | --- | --- | --- |
|  | **Tumour site** | | | | | | | | | | | |
|  | **Breast cancer** | | | **Prostate cancer** | | | **Colorectal cancer** | | | **Lung cancer** | | |
|  | **Yes** | **No** | **Std diff** | **Yes** | **No** | **Std diff** | **Yes** | **No** | **Std diff** | **Yes** | **No** | **Std diff** |
| Number of patients | 3,216 | 97,768 | - | 5,118 | 111,318 | - | 3,199 | 209,086 | - | 1,334 | 209,086 | - |
| Age | 80.2 | 80.2 | 0.000 | 81.2 | 81.2 | 0.000 | 81.8 | 81.8 | 0.000 | 78.0 | 78.0 | 0.000 |
| Sex |  |  |  |  |  |  |  |  |  |  |  |  |
| Male | 0.0% | 0.0% | 0.000 | 100.0% | 100.0% | 0.000 | 63.0% | 63.0% | 0.000 | 59.2% | 59.2% | 0.000 |
| Female | 100.0% | 100.0% | 0.000 | 0.0% | 0.0% | 0.000 | 37.0% | 37.0% | 0.000 | 40.8% | 40.8% | 0.000 |
| Ethnicity |  |  |  |  |  |  |  |  |  |  |  |  |
| Unknown | 39.3% | 39.3% | 0.000 | 36.4% | 36.4% | 0.000 | 35.8% | 35.8% | 0.000 | 37.1% | 37.1% | 0.000 |
| Black | 0.9% | 0.9% | 0.000 | 2.1% | 2.1% | 0.000 | 0.7% | 0.7% | 0.000 | 1.2% | 1.2% | 0.000 |
| Other | 3.3% | 3.3% | 0.000 | 3.4% | 3.4% | 0.000 | 3.8% | 3.8% | 0.000 | 2.3% | 2.3% | 0.000 |
| South Asian | 0.7% | 0.7% | 0.000 | 0.4% | 0.4% | 0.000 | 0.4% | 0.4% | 0.000 | 0.1% | 0.2% | 0.000 |
| White | 55.8% | 55.8% | 0.000 | 57.6% | 57.6% | 0.000 | 59.3% | 59.3% | 0.000 | 59.2% | 59.2% | 0.000 |
| **Pre-existing conditions** | | | | | | | | | | | | |
| IHD |  |  |  |  |  |  |  |  |  |  |  |  |
| No | 66.0% | 66.0% | 0.000 | 53.5% | 53.5% | 0.000 | 56.6% | 56.6% | 0.000 | 55.2% | 55.2% | 0.000 |
| Yes | 29.2% | 29.2% | 0.000 | 42.7% | 42.7% | 0.000 | 39.2% | 39.2% | 0.000 | 40.4% | 40.4% | 0.000 |
| Unknown | 4.8% | 4.8% | 0.000 | 3.8% | 3.8% | 0.000 | 4.2% | 4.2% | 0.000 | 4.4% | 4.4% | 0.000 |
| Valve disease |  |  |  |  |  |  |  |  |  |  |  |  |
| No | 72.4% | 72.4% | 0.000 | 74.9% | 74.9% | 0.000 | 73.6% | 73.7% | 0.000 | 76.5% | 76.5% | 0.000 |
| Yes | 22.4% | 22.4% | 0.000 | 20.8% | 20.8% | 0.000 | 21.9% | 21.9% | 0.000 | 19.2% | 19.2% | 0.000 |
| Unknown | 5.2% | 5.2% | 0.000 | 4.3% | 4.3% | 0.000 | 4.5% | 4.5% | 0.000 | 4.3% | 4.3% | 0.000 |
| Diabetes |  |  |  |  |  |  |  |  |  |  |  |  |
| No | 69.6% | 69.6% | 0.000 | 71.0% | 71.0% | 0.000 | 67.6% | 67.6% | 0.000 | 69.9% | 69.9% | 0.000 |
| Yes | 27.4% | 27.4% | 0.000 | 26.2% | 26.2% | 0.000 | 29.4% | 29.4% | 0.000 | 27.6% | 27.6% | 0.000 |
| Unknown | 3.0% | 3.0% | 0.000 | 2.8% | 2.8% | 0.000 | 3.0% | 3.0% | 0.000 | 2.5% | 2.5% | 0.000 |
| COPD |  |  |  |  |  |  |  |  |  |  |  |  |
| No | 81.4% | 81.4% | 0.000 | 78.8% | 78.8% | 0.000 | 79.7% | 79.7% | 0.000 | 56.3% | 56.3% | 0.000 |
| Yes | 13.6% | 13.6% | 0.000 | 16.4% | 16.4% | 0.000 | 15.8% | 15.8% | 0.000 | 39.4% | 39.4% | 0.000 |
| Unknown | 5.0% | 5.0% | 0.000 | 4.7% | 4.7% | 0.000 | 4.5% | 4.5% | 0.000 | 4.3% | 4.3% | 0.000 |

IHD= ischaemic heart disease. COPD= chronic obstructive pulmonary disease. Age is listed in years as mean (SD). All other information is number (%).

Propensity score includes the variables: age at HF admission, sex, ethnicity year of HF admission, IHD, valve disease, diabetes and COPD.

# **Supplementary Table 2.** Stage at cancer diagnosis by cancer site.

| **Stage** | **All sites** | **Site** | | | |
| --- | --- | --- | --- | --- | --- |
|  |  | **Breast** | **Prostate** | **Colorectal** | **Lung** |
| **1** | 2,202 (17.1%) | 797 (24.8%) | 592 (11.6%) | 411 (12.9%) | 402 (30.1%) |
| **2** | 2,223 (17.3%} | 894 (27.8%) | 568 (11.1%) | 603 (18.9%) | 158 (11.8%) |
| **3** | 1,279 (9.9%) | 187 (5.8%) | 417 (8.2%) | 455 (14.2%) | 220 (16.5%) |
| **4** | 1,098 (8.5%) | 80 (2.5%) | 664 (13.0%) | 147 (4.6%) | 207 (15.5%) |
| **Unknown** | 6,065 (47.1%) | 1,258 (39.1%) | 2,877 (56.2%) | 1,583 (49.5%) | 347 (26.0%) |
| **All stages** | 12,867 (100%) | 3,216 (100.0%) | 5,118 (100.0%) | 3,199 (100.0%) | 1,334 (100.0%) |
|  |  |  |  |  |  |
| **Follow-up recorded, n** | 12,116 | 3,045 | 4,816 | 3,014 | 1,241 |
| **Years, median (IQR)** | 1.56 (0.33, 4.29) | 2.00 (0.44, 5.10) | 1.61 (0.35, 4.38) | 1.53 (0.36, 4.16) | 0.77 (0.18, 2.30) |

n (column %). Lung cancer includes trachea, bronchus, and lung cancers.

IQR – interquartile range

# **Supplementary Figure 1:** Percentage point differences in receiving HF discharge medication between patients with and without cancer who had HFrEF and were discharged alive (N = 114,001).

Standardised across age at admission, year of admission, sex, ethnicity (White, Black, South Asian, Other, Unknown), and the following pre-existing diseases: valve disease, ischaemic heart disease, diabetes, chronic obstructive pulmonary disease. HF = Heart failure. ACEi/ARB = Angiotensin converting enzyme inhibitors or angiotensin receptor antagonists. MRA = mineralocorticoid receptor antagonists.

We excluded patients if they died in hospital or they had HFpEF.

# **Supplementary Figure 2:** Percentage point differences in receiving HF discharge medication between patients with different cancer characteristics and patients without cancer who had HFrEF and were discharged alive (N = 114,001). Patients with cancer split by lung cancer, non-lung cancer diagnosed ≤1 year, non-lung cancer diagnosed >1 and ≤3 years, and non-lung cancer diagnosed >3 years before HF hospital admission.

Regression adjustment used. Adjusted for age at admission, year of admission, sex, ethnicity (White, Black, South Asian, Other, Unknown), and the following pre-existing diseases: valve disease, ischaemic heart disease, diabetes, chronic obstructive pulmonary disease and standardised to whole cancer population. HF = Heart failure. ACEi/ARB = Angiotensin converting enzyme inhibitors or angiotensin receptor antagonists. MRA = mineralocorticoid receptor antagonists.

# **Supplementary Figure 3:** Adjusted all-cause survival by lung cancer patients, non-lung cancer patients ≤1 year from diagnosis, non-lung cancer patients >1 and ≤3 years from diagnosis and non-lung cancer patients >3 years from diagnosis, compared to patients without cancer

Adjusted for age at admission, year of admission, sex, ethnicity (White, Black, South Asian, Other, Unknown), and the following pre-existing diseases: valve disease, ischaemic heart disease, diabetes, chronic obstructive pulmonary disease. Standardised to characteristics of cancer population.

N=211,224 patients who were discharged alive and with available post-discharge follow-up.
